# Imaging and management of a small cell lung cancer metastasis/adrenal adenoma collision tumor: a case report and review of the literature

**DOI:** 10.1186/1477-7819-12-45

**Published:** 2014-02-26

**Authors:** Brian R Untch, Jinru Shia, Robert J Downey, Jorge A Carrasquillo, David M Panicek, Vivian E Strong

**Affiliations:** 1 Department of Surgery, Memorial Sloan-Kettering Cancer Center, 1275 York Ave, New York, NY 10065, USA; 2 Department of Pathology, Memorial Sloan-Kettering Cancer Center, New York, NY, USA; 3 Department of Radiology, Memorial Sloan-Kettering Cancer Center, New York, NY, USA

**Keywords:** Collision tumor, Small cell lung cancer, Adrenal adenoma, Fluorodeoxyglucose positron emission tomography, Adrenal mass

## Abstract

**Objective:**

We report a case of an adrenal collision tumor composed of a small cell lung carcinoma metastasis and a benign adrenal adenoma identified preoperatively on FDG-PET, CT and MRI and confirmed pathologically.

**Methods:**

The patient’s history, preoperative imaging characteristics, postoperative course, and histopathology are described. A review of the literature addressing adrenal collision tumors is provided.

**Results:**

A 47-year-old female was found to have a left upper lobe lung mass and an adrenal lesion on imaging. FDG-PET, CT and MRI of the adrenal suggested a metastatic lesion adjacent to an adrenal adenoma. CT-guided biopsy of the adrenal gland was consistent with a small cell lung cancer metastasis. The patient underwent systemic chemotherapy and had complete resolution of the left upper lobe mass. Post-treatment FDG-PET demonstrated a persistently enlarged adrenal gland with decreased but persistent FDG uptake. The patient underwent adrenalectomy and pathologic examination demonstrated a small cell lung cancer/adenoma collision tumor.

**Conclusions:**

This case and a review of the literature demonstrate that FDG, CT and MR imaging can all characterize the separate components of collision tumors within the adrenal gland.

## Background

Composite tumors are those that have two different histologic types intermixed, whereas collision tumors are those in which two tumors abut each other or are in close proximity [1]. Collision tumors have been reported in nearly every organ in the body. They may be composed of two primary tumors from the same organ, or a primary tumor and a metastasis. The close approximation of two malignancies can create diagnostic and therapeutic dilemmas. If a mixed benign and malignant collision tumor is biopsied and only the benign component identified, sub-optimal treatment may be delivered.

Here we report an unusual case of a patient noted to have two left adrenal lesions on imaging, which were correctly characterized radiologically as a metastasis adjacent to a benign adrenal adenoma. This was confirmed by fine needle aspirate cytology and pathology after adrenalectomy. To our knowledge this is the first reported case of a small-cell lung carcinoma and adrenal adenoma collision tumor.

## Case presentation

**Figure 1:**
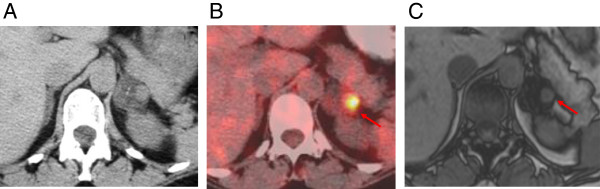
**Pretreatment imaging of an adrenal collision tumor. (A)** Non-contrast axial computed tomography scan (CT) demonstrating two distinct lesions in the left adrenal gland: a low attenuation 2.9 × 3.1-cm lesion as well as a higher attenuation 1.2 × 1.4-cm nodule, suspicious for metastatic disease. **(B)** Fused CT/fluorodeoxyglucose (FDG) positron emission tomography scan demonstrates an FDG avid lesion in the high attenuation component (standardized uptake value (SUVmax) 7) and no significant uptake in the low attenuation component. **(C)** Opposed-phase axial magnetic resonance imaging shows loss of signal intensity in the majority of the left adrenal mass, consistent with a lipid-rich adrenal adenoma, largely surrounding a 1.2-cm mass within the lateral portion of the adrenal gland that did not lose signal intensity, suggestive of metastatic disease.

**Figure 2:**
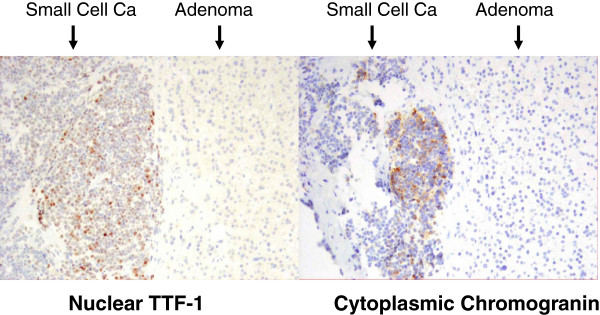
**Immunohistochemistry of resected collision tumor.** Nuclear thyroid transcription factor-1 (TTF-1) immunohistochemical staining (left) and cytoplasmic chromogranin staining (right) were positive in the small cell carcinoma component, but not in the adrenal adenoma.

A 47-year-old woman developed cough and hoarseness that persisted after a course of antibiotics. A computed tomography (CT) scan of the chest revealed a 6.0-cm mass in the left upper lobe of the lung and bulky hilar adenopathy. In the left adrenal, two lesions were identified on non-contrast-enhanced images: a low-attenuation 2.9 × 3.1-cm lesion suggestive of a lipid-rich adenoma, and a higher attenuation 1.2 × 1.4-cm nodule suspicious for metastatic disease (Figure 1A). Fluorodeoxyglucose positron emission tomography (FDG-PET) confirmed that the low attenuation lesion of 5 Hounsfield units was not hypermetabolic. The standardized uptake value based on body weight (SUVmax) was 1.8, consistent with an adenoma. The smaller, higher attenuation lesion (31 Hounsfield units) within the adrenal was hypermetabolic (SUVmax 7) consistent with metastatic disease (Figure 1B). At chemical-shift magnetic resonance imaging (MRI), two distinctly different components were demonstrated, also suggestive of a collision tumor (Figure 1C). A percutaneous CT-guided biopsy of the adrenal tumor revealed malignant cells consistent with small-cell lung cancer. After treatment with six courses of etoposide and cisplatin, her CT scan showed a marked decrease in the size of the lung tumor as well as near complete resolution of the associated lymphadenopathy. However, the left adrenal remained persistently enlarged on CT, despite a decrease in FDG avidity on PET imaging (SUVmax decreased from 7.0 to 2.7). Given the patient’s age and response to chemotherapy, adrenalectomy was recommended to determine the presence of persistent disease after chemotherapy, and possibly to render the patient free of metastases. Lung resection was not thought to be beneficial because of the tumor response noted on imaging. The patient underwent an uncomplicated laparoscopic left adrenalectomy. Pathologic examination of the adrenal revealed a small-cell lung carcinoma metastasis adjacent to an adrenal adenoma. Nuclear thyroid transcription factor-1 (TTF-1) and cytoplasmic chromogranin immunohistochemical staining were positive in the small-cell carcinoma component, but not in the adrenal adenoma (Figure 2). Postoperatively the patient underwent a further three months of chemotherapy.

The patient remained free of disease for 1 year, but was then found to have pancreatic metastases. She died 4 months later, approximately two years after her original diagnosis.

## Conclusions

The reason why collision tumors develop is unclear. Malignancies have been found to promote local and distant sites of disease, and it is plausible that these same factors could facilitate the growth of second primary tumors. The initial tumor may create a milieu that makes it easier for a second tumor to grow [2,3]; *in vitro* and clinical evidence suggests that peri-tumoral tissue can facilitate the growth of tumor cells in and around the primary tumor via paracrine signaling [4,5]. Primary tumors are capable of secreting circulating growth factors that can facilitate metastatic spread and subsequent metastatic tumor growth [6]. Also playing a role may be bone marrow-derived cells which, when recruited to tumor sites, can facilitate growth of the primary tumor and metastases [7].

Collision tumors are rare in the adrenal gland at imaging, occurring in 2 out of 104 patients MRI in a study by Schwartz *et al*. [2]. Case reports of biopsy-proven adrenal collision tumors in the literature are summarized in Table 1. In all cases, primary adrenal tumors were one component of the collision tumor [2,8-13]. In 9 of 11 patients, the second tumor was a metastasis. Identification of the tumors by imaging was successful in all but one case [8]. This case was a large heterogeneous tumor that was composed of a rectal cancer metastasis and an adrenal carcinosarcoma. Otherwise, CT and MRI were able to successfully demonstrate that two different tumor types were present in the same adrenal gland. In this case the successful imaging identification of a collision tumor led to appropriate biopsy of a metastasis and subsequent systemic therapy.

**Table 1:** Published reports of biopsy-proven adrenal collision/composite tumors

**Author**	**Histology of first tumor**	**Histology of second tumor**	**Imaging modality**	**Imaging results**	**Lesion size**
Bertolini *et al*. [8]	Adrenal carcinosarcoma	Rectal cancer	CT/MRI	One heterogeneous tumor	14 cm
Thorin-Savoure *et al*. [9]	Adrenal adenoma	Sigmoid cancer	CT	Two components of different attenuation	3 cm
Thorin-Savoure *et al*. [9]	Adrenal adenoma	Breast cancer	CT	Two components of different attenuation	4.1 cm
Hagspiel [10]	Adrenal myelolipoma*	Hodgkin’s lymphoma*	CT	Two components of different attenuation	1.2. cm
Blake *et al*. [11]	Adrenal adenoma	Spermatic cord leiomyoscarcoma	PET/CT	Two components of different attenuation and metabolic activity	2.5 cm
Otal *et al*. [12]	Adrenal adenoma	Adrenal myelolipoma	CT/MRI	Two components of different attenuation and signal intensity	NA
Otal *et al*. [12]	Adrenal adenoma	Adrenal myelolipoma	CT	Two components of different attenuation	NA
Schwartz *et al*. [2]	Adrenal adenoma	Non-small cell lung cancer	MRI	Two components of different signal intensity	NA
Schwartz *et al*. [2]	Adrenal adenoma	Breast cancer	MRI	Two components of different signal intensity	NA
Hoshi *et al*. [13]	Adrenal adenoma*	Non-small-cell lung cancer*	CT	Two components of different attenuation	NA
Untch *et al*. (Current study)	Adrenal adenoma	Small-cell lung cancer	CT/PET/MRI	Two components of different attenuation, signal intensity, and metabolic activity	2.5 cm

*Histology confirmed at autopsy. CT, computed tomography; MRI, magnetic resonance imaging; PET, positron emission tomography; NA – not available.

Various radiologic techniques are available for characterization of adrenal masses discovered incidentally during imaging performed for other purposes [14]. Approximately 7 to 10% of such masses represent adrenal adenomas, which can be identified on non-contrast CT if their attenuation measures less than 10 Hounsfield units, or by the more sensitive quantitative changes in enhancement values obtained at baseline and at 15-minute washout CT. Alternatively, chemical-shift imaging can identify about 70% of adrenal adenomas as such, based on their high lipid content. On opposed-phase images, the signal intensities of the intermixed lipid and water components present within an adenoma will cancel each other, resulting in signal loss in those locations relative to the in-phase images. At PET scanning, adrenal adenomas generally show low FDG avidity. In retrospect, the patient in this case report could have been managed with a combined PET/CT alone as this also allows for interval evaluation of the tumor metabolic activity after systemic therapy.

The decision to pursue adrenalectomy for this patient was based on the patient’s age, the excellent treatment response, and the persistent adrenal lesion noted on CT that had a discordant FDG-PET response compared to the primary site of disease. Additionally, the literature supports the possible benefit of resection of a solitary site of hematogenous metastasis in non-small-cell lung cancer [15]. The efficacy and safety of laproscopic adrenalectomy has made metastasectomy a favored approach for patients with isolated metastatic disease. The laparoscopic approach is associated with shorter hospital stay, lower blood loss and lower morbidity [16,17] This, combined with the five-year survival data ranging from 24 to 31%, depending on the tumor type, makes the treatment approach reasonable in select patients [18,19].

In summary, we report the first case in the literature of an adenoma-small-cell lung cancer collision tumor in the adrenal gland. This case and the literature review demonstrate that FDG-PET, CT and MRI can all distinguish two separate components of collision tumors within the adrenal gland and, in the case of adrenal adenoma, provide a definitive diagnosis for that component. When a satisfactory post-treatment evaluation of such lesions is not possible with these imaging modalities, surgical intervention in select patients should be considered.

## Consent

Written informed consent was obtained from the patient for publication of this Case report and any accompanying images. A copy of the written consent is available for review by the Editor-in-Chief of this journal.

## Abbreviations

CT: Computed tomography; MRI: Magnetic resonance imaging; FDG-PET: Fluorodeoxyglucose positron emission tomography; SUV: Standardized uptake value; TTF-1: Thyroid transcription factor-1.

## Competing interests

The authors declare that they have no competing interests.

## Authors’ contributions

BRU performed the literature review, and designed and drafted the manuscript. JS, RJD, JAC, and DMP assisted in drafting the manuscript and designing the figures. VES conceived the study and assisted in drafting the manuscript. All authors read and approved the final manuscript.
